# Childbirth hospitalizations in Bipolar disorder patients: a nationwide study protocol

**DOI:** 10.1192/j.eurpsy.2022.1958

**Published:** 2022-09-01

**Authors:** G. Araújo, M. Gonçalves-Pinho, A.R. Ferreira, L. Fernandes, A. Freitas

**Affiliations:** 1 University of Porto, Faculty Of Medicine, Porto, Portugal; 2 CINTESIS - Center for Health Technology and Services, Faculty of Medicine, University of Porto, Department Of Community Medicine, Information And Health Decisions Sciences, Porto, Portugal; 3 Centro Hospitalar do Tâmega e Sousa, Departamento De Psiquiatria E Saúde Mental, Penafiel, Portugal; 4 Faculty of Medicine, University of Porto, Department Of Community Medicine, Information And Health Decision Sciences (medcids), Porto, Portugal; 5 Faculty of Medicine, University of Porto, Department Of Clinical Neurosciences And Mental Health, Porto, Portugal; 6 Psychiatry Service, Centro Hospitalar Universitário De São João, Porto, Portugal

**Keywords:** Childbirth, Hospitalizations, Pregnancy, bipolar disorder

## Abstract

**Introduction:**

Bipolar disorder (BD) is usually diagnosed in adulthood, around childbearing age. Research has shown that BD has deleterious effects on pregnant women and birth outcomes. However, few nationwide studies using administrative data have approached this at-risk population focusing specifically on childbirth.

**Objectives:**

This study aims to characterize hospitalizations of women with BD in the peripartum period regarding sociodemographic and clinical variables and to investigate the impact BD has on hospitalization outcomes.

**Methods:**

An observational retrospective study will be performed using an administrative database that comprises routinely collected hospitalization data from all mainland Portuguese public hospitals. International Classification of Diseases, Ninth Revision, Clinical Modification (ICD-9-CM) codes will be used to identify all women’s admissions for childbirth purposes (V27.X) and codes 296.XX (except 296.2X, 296.3X, 296.9X) will be used to ascertain BD. Episodes will be assigned to one of two mutually exclusive groups (with vs without BD). Multivariate methods will be used to compare both groups concerning key variables and outcomes. This work will comply with the RECORD statement recommendations.

**Results:**

Descriptive and analytical statistics will be conducted in order to describe and characterize this group of patients. Results will be presented as crude and adjusted odds ratio quantifying the risk associated with BD in pregnancy, childbirth and hospitalization outcomes. Findings will be disseminated via publication in peer-reviewed journals.

**Conclusions:**

With this nationwide analysis, we expect to contribute to a better understanding of the demographic and clinical profile of pregnant women with BD and to encourage timely medical and psychological interventions during gestation and childbirth.

**Disclosure:**

No significant relationships.

